# Hypnotic use in a population-based sample of over thirty-five thousand interviewed Canadians

**DOI:** 10.1186/1478-7954-4-15

**Published:** 2006-11-24

**Authors:** Aliya Kassam, Scott B Patten

**Affiliations:** 1 Section of Community Mental Health, Health Services Research Department, Institute of Psychiatry, King's College London, SE5 8AF, UK; 2 Department of Community Health Sciences, University of Calgary, 3330 Hospital Drive NW, T2N 4N1, Calgary, AB, Canada; 3 Department of Psychiatry, University of Calgary, 1403 - 29 Street NW. Calgary, T2N 2T9, Canada

## Abstract

**Background:**

As with most medications, benzodiazepine and similar sedative hypnotics (BDZ/SSH) can produce both beneficial and adverse effects. Pharmacoepidemiological studies have been limited in their capacity to evaluate the relationship between these medications and psychiatric diagnoses in non-clinical populations. The objective of this study was to provide a description of the pattern of use of BDZ/SSH medications in relation to both demographic and diagnostic data in a community population.

**Methods:**

The source of data for this study was the Canadian Community Health Survey (CCHS 1.2), also known as the Canadian National Study of Mental Health and Well-being. This study was based on a nationally representative sample that included over 35 thousand subjects with a response rate of 77%. The survey interview included the latest version of the Composite International Diagnostic Interview (CIDI), which was developed for the World Health Organization's WHO Mental Health 2000 project. Current medication use was also recorded.

**Results:**

As expected, BDZ/SSH use was more common in women than in men (4.2%, 95% CI 3.9 to 4.6 vs. 2.5%, 95% CI 2.2 to 2.8) and its frequency increased with age, 8.5% (95% CI 7.7 to 9.4) of those over the age of 65 compared to 2.4% (95% CI 2.2 to 2.7) of those aged 18 to 64 years. These medications were more frequently used in subjects with low levels of education (4.8%, 95% CI% 4.3 to 5.2) vs. high levels of education (2.4%, 95% CI 2.1 to 2.6) and low personal incomes (5.7%, 95% CI 5.2 to 6.3) vs. high personal incomes (2.3%, 95% CI 2.0 to 2.6). BDZ/SSH use was strongly associated with the presence of mood or anxiety disorders, but not with substance use disorders. Demographic differences persisted after statistical adjustment for diagnosis.

**Conclusion:**

The observation that benzodiazepine use is more frequent in women, increases with age and is higher in low income and education groups supports previous findings. These results help to confirm that these differences are not accounted for by psychiatric diagnoses.

## Background

Benzodiazepines are in their fifth decade of use and remain among the most frequently prescribed drugs. These medications can be beneficial in the short-term management of diverse symptoms, most notably anxiety, insomnia and agitation. However, they also have adverse effects which can be fatal, particularly with long-term use and use in the elderly, where epidemiological studies have linked benzodiazepine exposure to motor vehicle accidents [1], hip fractures [2,3] cognitive problems and self care limitations [4]. The increased risk of injury due to traffic accidents may not be restricted to the elderly age group [5-7]. Benzodiazepine use can lead to dependence [8,9], and can contribute to overdose fatalities [10].

Zopiclone and zaleplon are non-benzodiazepine sedative hypnotics, which nevertheless share many pharmacological properties with benzodiazepines, potentially including many of their adverse effects and risks [11-15]. In this paper, benzodiazepines and zopiclone are grouped together as benzodiazepines and similar sedative hypnotics (BDZ/SSH). At the time of the study zaleplon had only been recently approved in Canada and this medication had not yet been assigned a unique identifying code, it was therefore not included in the analysis. Another drug, zolpidem was not included, as it had not been approved for use in Canada when this study was conducted.

An increased frequency of BDZ/SSH use with age has been reported [16-18], a phenomenon that cannot be due to confounding by mood or anxiety disorder status, since the prevalence of these disorders declines with age [19,20]. However, these same studies have reported a higher frequency of use in women, which could result from a higher prevalence of mood and anxiety disorders in women. Similarly, if subject characteristics indicative of low socioeconomic status (e.g. lower levels of education, low income) and unmarried status are associated with mood and anxiety disorders, these variables could appear to be associated with BDZ/SSH use because of confounding with mood or anxiety disorders. The only recent study to evaluate mood and anxiety disorder status in relation to current BDZ/SSH use in a general population sample was a French study [17] that included a brief diagnostic interview, the Mini International Neuropsychiatric Interview (MINI) [21]. The authors reported that the association of BDZ/SSH use with increasing age and female sex persisted after adjustment for mood and/or anxiety disorder status. The objective of this analysis was to extend these observations using data from a 2002 Canadian survey called the Canadian Community Health Survey 1.2 (CCHS 1.2), or the Canadian National Survey of Mental Health and Well-being. This study included a more detailed diagnostic assessment interview than the MINI instrument, the WHO Mental Health Version of the Composite International Diagnostic Interview (WMH-CIDI) [22].

## Methods

The CCHS 1.2 was a cross-sectional survey conducted by Statistics Canada (the Canadian Government's statistical agency) to collect information on mental health status, mental health care utilization and mental health determinants. The mental disorders investigated included depression, mania, panic disorder, social phobia, agoraphobia, alcohol dependence and illicit drug dependence. These were determined using a version of the CIDI developed for the World Mental Health 2000 Survey (WMH2000) [22]. An exception was alcohol dependence, for which the CCHS 1.2 used a CIDI-Short Form (CIDI-SF) [23]. Both the WMH2000 CIDI and CIDI-SF interviews applied definitions from the Diagnostic and Statistical Manual of Mental Disorders, Fourth Edition (DSM-IV) [8].

The WMH2000 version of the CIDI generates past year and lifetime diagnoses of psychiatric disorders. During the interview, which was usually conducted in their homes, study participants were asked to collect all of their medication containers for any medication taken in the preceding two days. These were subsequently coded for analysis using the WHO Anatomic Therapeutic Classification (ATC) system. Data were collected between May 2002 and December 2002.

Education was categorized at two levels, subjects with less than high school education and subjects having graduated from high school, with or without additional post-secondary schooling. Income was designated as "low" using Statistics Canada formulas that account for total family income, adjusted for family size. The "lowest" and "low middle" categories were aggregated for analysis.

The analysis presented here consisted of tabulation and logistic regression analyses. Design effects were accounted for by the use of sampling weights and a bootstrap procedure for variance estimation. All analyses were conducted using SAS statistical software [24]. The study received approval from the University of Calgary Conjoint Medical Ethics Review Board.

## Results

The response rate for the CCHS 1.2 was 77%. For this analysis, the total sample (n = 36,984) was restricted to include 35,236 subjects 18 years old or older. The restricted sample comprised 15,889 (45.1%) men and 19,347 (54.9%) women. The overall (weighted) frequency of use of BDZ/SSH was 3.4% (95% CI 3.1 – 3.6). Table 1 shows the frequency of benzodiazepine use in relation to age, sex, marital status, income and education.

**Table 1:** Frequency (%) of Benzodiazepine and Similar Sedative-Hypnotic Use, by Demographic Variables

**Demographic Variables**	**%**	**95% C.I.**
**Sex**		
• Men	2.5	2.2 – 2.8
• Women	4.2	3.9 – 4.6
		
**Age**		
• 18 to 64 years	2.4	2.2 – 2.7
• 65 years and over	8.5	7.7 – 9.4
		
**Marital Status**		
• Married or Common-law	2.8	2.5 – 3.1
• Single, Separated, Divorced, Widowed	4.4	4.0 – 4.8
		
**Income**		
• High Income	2.3	2.0 – 2.6
• Low Income	5.7	5.2 – 6.3
		
**Education**		
• High Education	2.4	2.1 – 2.6
• Low Education	4.8	4.3 – 5.2

The frequency of use, as shown in Table 1, was higher in women, those over the age of 65, those with low education, low income and those who were single, separated, divorced or widowed.

Frequency estimates stratified for disorder status are presented in Table 2. After stratifying for the presence of anxiety or mood disorders, the pattern of an increased frequency of benzodiazepine and similar sedative-hypnotic use in women, unmarried subjects, and those with low income and education continued. As expected, anxiety and mood disorders were themselves strongly associated with benzodiazepine and similar sedative-hypnotic use.

**Table 2:** Frequency (%) of Benzodiazepine and Similar Sedative-Hypnotic Use in Demographic Categories, Stratified by the Presence of an Anxiety or Mood Disorder

	**%**	
	**With Anxiety/Mood Disorder**	**Without Anxiety/Mood Disorder**
**Sex**		
• Men	9.4	2.0
• Women	11.0	3.3
		
**Marital Status**		
• Married or Common-law	10.0	2.3
• Single, Separated, Divorced, Widowed (Unmarried)	10.9	3.4
		
**Income**		
• High Income	8.5	1.8
• Low Income	14.1	4.6
		
**Education**		
• High Education	8.0	1.8
• Low Education	13.9	3.8

Logistic regression models predicting benzodiazepine or similar sedative-hypnotic use were generated using sex, marital status, income or education, the presence of an anxiety or mood disorder and the corresponding two-way interaction term as predictor variables. Wald tests identified no evidence of interaction between these variables and anxiety or mood disorders in the models containing sex, marital status or education. In the model for income, however, the presence of an anxiety or mood disorder and low income interacted according to the Wald test (*z *= 4.7, *p *= 0.03), indicating that BDZ/SSH use was more strongly associated with (low) income in subjects without a mood or anxiety disorder (OR 2.7) than in subjects with a mood or anxiety disorder (OR 1.8). After adjustment for the presence of an anxiety or mood disorder, the odds ratio for (female) sex was 1.6 (95% CI 1.3 – 1.8). For (unmarried) marital status, the odds ratio was 1.4 (95% CI 1.2 – 1.6) and for (less than high school) education was 2.1 (95% CI 1.8 – 2.5).

Table 3 shows the frequency of benzodiazepine or similar sedative-hypnotic use in relation to clinical diagnoses as well as other chronic illnesses that were recorded during the CCHS 1.2 interview as self-report (rather than CIDI-based) items. The highest frequency is in those with CIDI diagnosed agoraphobia (with or without panic) as well as those with self-reported schizophrenia. Those with self-reported epilepsy also reported a high frequency of use. The lowest frequency of benzodiazepine or similar sedative-hypnotic use occurred in those with substance use disorders.

**Table 3:** Frequency (%) of Benzodiazepine and Similar Sedative-Hypnotic Use, by Diagnosis

**Diagnosis**	**%**	**95% C.I.**
Any Mood disorder	11.9	10.0 – 13.8
Any Anxiety disorder	11.4	9.4 – 13.4
Any Substance abuse disorder	2.3	1.4 – 3.3
Agoraphobia with panic*	17.5	10.7 – 24.4
Agoraphobia without panic	17.7	9.0 – 26.5
Alcohol Dependence	2.2	1.1 – 3.2
Mania	10.3	6.1 – 14.5
Panic Disorder*	14.2	10.4 – 17.9
Major Depressive Episode	12.3	10.3 – 14.3
Social Phobia	10.0	7.7 – 12.4
Self-reported Epilepsy**	12.4	6.4 – 18.4
Self-reported Schizophrenia**	19.2	11.2 – 27.1

* The diagnoses "agoraphobia with panic" and "panic disorder" are not mutually exclusive.

**The interview included items asking about a diagnosis of these conditions "by a health professional."

## Conclusion

The pattern of association between BDZ/SSH use and demographic variables resembles the pattern reported in recent French and Italian studies evaluating current use [17,18], but the actual frequency of use was much lower in the Canadian sample. In the French study, the frequency of current use was 7.5% [17] and in the Italian study past week use was 8.6% [18]. European studies examining use over longer periods have produced only slightly higher estimates, for example, 9.8% over the past 12-months in the ESEMeD survey [20]. These results are consistent with the idea that a large proportion of BDZ users take the medications for long periods.

Lagnaoui et al. [17] were the only previous investigators to examine the persistence of associations between BDZ/SSH use and demographic variables after adjustment for the presence of a mood or anxiety disorder. Their study included the MINI [21] interview and found, as did the current study, that these disorders were strongly associated with the use of BDZ/SSH, but also that inclusion of these diagnoses in a regression analysis did not eliminate the effect of demographic variables.

Although the CCHS 1.2 provided an opportunity to describe the pattern of use of BDZ/SSH, the epidemiological determinants of BDZ/SSH were not necessarily comprehensively evaluated by the interview used in the study. Relevant determinants may include psychiatric symptoms that are not related to these disorders, or disorders that were not covered by the interview. For example, subjects with inadequate financial resources may experience more stress, worry or insomnia than those with higher income. A higher frequency of use in subjects with such symptoms, in the absence of a mood or anxiety disorder, could account for the interaction reported here between diagnosis and income category. Some of the differences may be related to health care utilization patterns, rather than to clinical or demographic factors. The increased prescribing for women may, for example, be related to an increased propensity to seek treatment. Alternatively, demographic differences in the frequency of use may be due to prescriber characteristics.

BDZ/SSH use was found to occur with a higher frequency in subjects with anxiety or mood disorders, but not in subjects with substance use disorders. Thus, the frequency of use seems to be more related to clinical and demographic factors than to dependence and abuse, at least when these substance-use disorders are categorized in relation to DSM-IV criteria.

One weakness of this study was the version of the ATC classification used. Statistics Canada used a version that did not include zaleplon, which may therefore have been coded into an "other" category. As a result, the frequency of BDZ/SSH use may have been underestimated. However the size of the residual or unspecified sedative-hypnotic category was small suggesting that the extent of bias would not be large. Some older sedative-hypnotics (e.g. chloral hydrate, barbiturates) were used too infrequently to be included in the analysis. As the data source was a population survey of household residents, homeless and institutionalized subjects were excluded. The results cannot be generalized to these other populations.

Certain demographic variables are associated with the use of BDZ/SSH medications, yet this does not appear to be easily accounted for by major categories of mental disorder. Therefore, focused clinical studies should be conducted in order to identify the causes of these differences, and to clarify their clinical significance since BDZ/SSH use in those without obvious indications may also point to extensive misuse.
